# Peripheral Nerve Stimulation to the Transverse Abdominis Plane as Therapy for Refractory Abdominal Pain: A Case Series of Two Patients

**DOI:** 10.7759/cureus.97686

**Published:** 2025-11-24

**Authors:** Christopher J Mallard, Preston C Rippe, Madhur Batra, Michael E Harned

**Affiliations:** 1 Anesthesiology, University of Kentucky, Lexington, USA; 2 Physical Medicine and Rehabilitation, University of Kentucky, Lexington, USA; 3 Physical Medicine and Rehabilitation, Spaulding Rehabilitation Hospital, Harvard Medical School, Boston, USA

**Keywords:** abdominal wall pain, chronic abdominal pain, chronic pain management, neuromodulation, peripheral nerve stimulation, refractory pain, transversus abdominis plane, ultrasound-guided procedures

## Abstract

Peripheral nerve stimulation (PNS) is an emerging therapeutic intervention for chronic pain that modulates peripheral nerve activity, improving the interplay between Aβ and C fibers to reduce central sensitization and local nociceptive signaling. While the use of PNS is well supported for extremity neuropathic pain, evidence for its application in the transverse abdominis plane (TAP) remains limited. We present two patients with chronic refractory abdominal pain who underwent ultrasound-guided TAP stimulation using the SPRINT PNS (SPR Therapeutics; Cleveland, OH) system. Both experienced greater than 90% pain reduction during the 60-day stimulation period and at three-month follow-up, with one patient reporting sustained benefit beyond five months. No complications occurred other than a transient lead displacement in one case. These cases suggest that TAP PNS may be a safe and effective option for refractory abdominal pain and support further prospective evaluation of this novel application.

## Introduction

Chronic abdominal pain syndromes, which include ilioinguinal neuralgia and postherpetic neuralgia (PHN) of the abdominal wall, are associated with significant epidemiological and economic burden. Ilioinguinal neuralgia is the most reported postsurgical pain condition following lower abdominal surgeries such as hernia repair, appendectomy, or gynecologic surgery. Rates have been reported to affect up to 10% of patients and can lead to persistent pain and functional impairment [1,2]. The annual rate of PHN in the US is approximately 57.5 cases per 100,000 person-years, with an estimated 114,000 new cases each year among adults [3].

Peripheral nerve stimulation (PNS) is an emerging therapeutic intervention for chronic pain. PNS is thought to modulate peripheral nerve activity, resulting in improved interplay between Aβ fibers and C fibers, leading to decreased central sensitization and local nociceptive signaling [4]. Sites with the greatest level of evidence for PNS include the peripheral nerves of the upper and lower extremities for the treatment of posttraumatic and postsurgical pain, complex regional pain syndrome, phantom limb pain, and hemiplegic shoulder pain. However, clinical evidence is currently limited regarding PNS to the transverse abdominis plane (TAP) for chronic refractory abdominal pain [5]. TAP blocks were first described as a means for perioperative and postoperative pain management, with current utilization in acute/regional anesthesia for their ability to provide local anesthetic to the plane between the transverse abdominis and internal oblique muscles. The TAP is a thin fascial layer between the internal oblique and transversus abdominis muscle. Cadaver studies have described that the anterior rami of the lower thoracic (T6-T12) and first lumbar (L1) spinal nerves can be found within the plane with extensive branching and communication. These segmental nerves eventually give rise to the subcostal, intercostal, ilioinguinal, and iliohypogastric nerves. Targeting the anterior rami within the TAP with local anesthetic, as described for TAP blocks, targets the entire anterolateral abdominal wall [6-8]. Despite the success of TAP blocks in the treatment of acute pain, there are limited data for the use of TAP PNS for refractory chronic abdominal pain.

In current practice, PNS can be delivered through several system designs. Like spinal cord stimulation (SCS), candidates typically undergo a trial period before consideration for permanent implantation. Several commercial manufacturers offer PNS systems, which vary in programming capabilities and hardware configuration. Most devices provide tonic stimulation (continuous, regular pulses), and many also offer alternative waveforms such as burst or high-frequency stimulation. System designs range from single-lead devices powered by external generators to multi-lead systems with fully implantable pulse generators. Temporary PNS systems are intended for short-term use, while others are designed for permanent implantation [9,10].

Current clinical evidence for TAP PNS includes a report from 2014 describing a patient who underwent placement of a 16-contact dorsal column percutaneous lead and an ultrasound-guided 8-contact stimulator lead to the TAP for abdominal pain following multiple abdominal surgeries, with reported consistent 80-90% pain reduction as well as a reduction of daily morphine milligram equivalents (MME) at the three-month follow-up visit [7]. An additional study from 2024 reviewed four patients who underwent multi-contact PNS placement for abdominal wall pain, with three of the four patients reporting sustained abdominal pain reduction and ultimately undergoing permanent PNS placement [11]. Current Best Practices Guidelines by the American Society of Pain and Neuroscience (ASPN) for PNS demonstrate six trunk/pelvic peripheral nerve targets appropriate for PNS, including the ilioinguinal and iliohypogastric nerves. These guidelines conclude that there is limited evidence (Level III, Grade C) to suggest that PNS may alleviate neuropathic pain syndromes involving the trunk [5]. Targeting the TAP allows access to the segmental nerves that provide sensory innervation to the anterolateral abdominal wall. Given the complexity and functional impact of chronic refractory abdominal pain, PNS delivered at this site may offer a viable option for long-term relief. Given the limited reports in the existing literature, we present two patients with chronic refractory abdominal pain who experienced substantial and lasting pain relief following TAP PNS. Informed consent was not obtained due to the retrospective nature of this chart review in accordance with institutional policy.

## Case presentation

A 49-year-old female with a medical history significant for lumbar radiculopathy, myofascial pain, irritable bowel syndrome, anxiety, depression, and diverticulosis presented with chronic bilateral ilioinguinal neuralgia. She described a constant, sharp, aching 8/10 visual analog scale (VAS) pain in the bilateral abdomen and groin that began in 2017. Her symptoms worsened with most activities of daily living and consistently improved with steroid-containing TAP blocks. The pain developed after a hysterectomy performed in 2018 for endometriosis and fibroids.

Her prior workup included gastroenterology and pelvic physical therapy consults. A CT angiogram of the abdomen and pelvis (April 2021) demonstrated patent abdominal vasculature with mild narrowing of the distal superior mesenteric vein (SMV) without associated inflammation. A CT of the abdomen and pelvis (March 2021) showed no pathologic abnormalities aside from a subtle anterior mesenteric nodule. A CT renal stone study (March 2021) demonstrated no urinary calculi and mild thickening of the vaginal wall suggestive of inflammation. A right upper-quadrant ultrasound (March 2021) was unremarkable. Magnetic resonance defecography (May 2019) revealed significant pelvic floor descent with rectocele and cystocele, along with submucosal edema of the sigmoid colon and rectum. Laboratory studies from May 2021 demonstrated a normal complete blood count and comprehensive metabolic panel. She reported prior normal esophagogastroduodenoscopy and colonoscopy results. She failed pelvic-floor physical therapy, and gastroenterology ultimately suspected a musculoskeletal etiology. Physical examination showed no abdominal distention or guarding and only generalized tenderness.

Her abdominal pain remained refractory to hydrocodone-acetaminophen 7.5-325 mg every six hours as needed, gabapentin 300 mg three times daily, pregabalin 50 mg twice daily, methocarbamol 750 mg three times daily, meloxicam 15 mg daily as needed, ibuprofen 800 mg twice daily, and acetaminophen 1000 mg every six hours as needed. She was on no pain medications at the time of the procedure. She also failed more than six weeks of physical therapy, aquatic therapy, and a home exercise program. Over the preceding three years, she underwent multiple unilateral and bilateral ilioinguinal/iliohypogastric nerve blocks with an average of 90% pain relief, though none lasted longer than two weeks.

**Figure 1:**
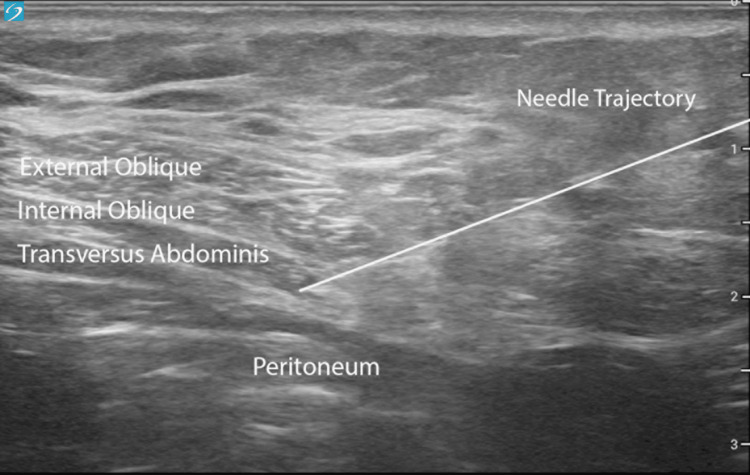
In-plane ultrasound visualization of the needle approach to the TAP during PNS lead placement TAP, transverse abdominis plane; PNS, peripheral nerve stimulation

Given persistent severe symptoms, she underwent bilateral ultrasound-guided placement of temporary PNS leads (SPRINT, SPR Therapeutics; Cleveland, OH) into the TAP. The patient was positioned prone and prepped in sterile fashion. A 15-4 MHz linear transducer was used to identify the TAP along the posterior axillary line. Using an in-plane approach, a 19-gauge stimulating probe was advanced through a 17-gauge percutaneous sleeve into the fascial plane between the internal oblique and transversus abdominis muscles to target the anterior rami of T6-L1. Test stimulation produced comfortable paresthesia over her painful abdominal and groin regions. The probe was exchanged for a 20-gauge Microlead Introducer containing the PNS lead, and proper stimulation coverage was reconfirmed. The introducer-sleeve assembly was withdrawn with manual traction to deploy the distal anchor within the TAP (Figure 1). Final programming consisted of dual-lead 100-Hz continuous stimulation, 24 hours per day, for the full 60-day trial. The patient reported immediate pain relief. No intraprocedural complications were noted, nor infection or local skin irritation for the lead or lead-securing system.

She experienced a greater than 90% reduction in right abdominal and groin pain throughout the trial, maintained at her 90-day follow-up visit. Her left lead was inadvertently displaced three weeks into the trial during a dressing change, though she reported more than 90% relief on the left side while the lead remained in place. A permanent PNS implant was offered, but declined due to the inability to afford the surgery center fee. She later elected to repeat bilateral TAP PNS and again reported more than 80% bilateral abdominal and groin pain reduction at both her two- and three-month follow-up visits, with reported VAS scores of 1-2/10. There are no charted quality-of-life measures to report for this patient.

An 80-year-old male presented with chronic right abdominal PHN with associated ilioinguinal neuropathic pain. He developed shingles in 2020 and initially reported his pain as a constant, aching, burning sensation rated 7/10, worsening throughout the day and exacerbated by prolonged standing and walking. His symptoms improved with activity pacing, rest, topical lidocaine, and hydrocodone. Prior to intervention, his Oswestry Disability Index (ODI) was 30, and his VAS pain score was 5/10. A CT angiogram of the abdomen performed in 2020 demonstrated bilateral triple-vessel runoff; critical stenosis of the distal right superficial femoral artery due to dense calcified plaque; mild to moderate stenosis of the left superficial femoral artery; mild proximal renal artery stenosis (left greater than right); and incidental chronic right lower-lobe atelectasis with a small associated pleural effusion.

His abdominal and neuropathic pain was refractory to extensive medication trials, including fentanyl 25 mcg/hour transdermal patches, gabapentin 800 mg three times daily, duloxetine 60 mg daily, naproxen 500 mg daily, topical 2% lidocaine gel, and lidocaine-prilocaine 2.5-2.5% cream. He additionally failed multiple interventional therapies, including right T11 and T12 transforaminal epidural steroid injections, a right T10-T12 dorsal root ganglion stimulation trial and implant, and a dorsal column SCS trial. At the time of PNS evaluation, he was taking hydrocodone-acetaminophen 5-325 mg, one-half tablet twice daily as needed, for persistent uncontrolled pain.

**Figure 2:**
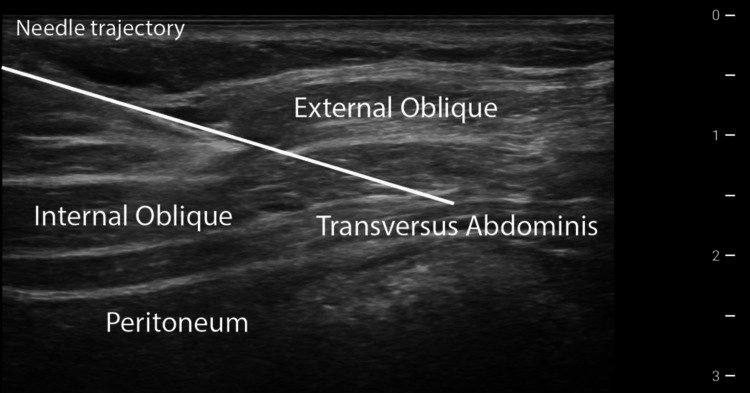
In-plane ultrasound visualization of the needle approach to the TAP during PNS lead placement TAP, transverse abdominis plane; PNS, peripheral nerve stimulation

He then underwent ultrasound-guided placement of a temporary peripheral nerve stimulator (SPRINT PNS System, SPR Therapeutics; Cleveland, OH) targeting the right TAP. The patient was positioned supine, prepped, and draped in sterile fashion. A 15-4 MHz linear ultrasound transducer was placed along the anterior axillary line to identify the TAP. Using an in-plane technique, a 19-gauge stimulating probe was advanced through a 17-gauge percutaneous sleeve into the fascial plane between the internal oblique and transversus abdominis muscles to target the anterior rami of T6-L1. Test stimulation elicited comfortable paresthesia covering the patient’s symptomatic region. The probe was removed and exchanged for a 20-gauge Microlead Introducer containing the PNS lead. Following reconfirmation of appropriate coverage, the introducer-sleeve assembly was withdrawn to deploy the distal anchor within the TAP (Figure 2). Final programming consisted of dual-lead continuous stimulation at 100 Hz, 24 hours per day, for the 60-day trial period.

The patient reported a greater than 90% reduction in right abdominal pain throughout the trial. His course was notable for a small area of “electric pain” in the lower abdomen during forward flexion, suspected to be secondary to an unrelated pannus yeast infection or minor lead position change with movement. No complications such as skin irritation, infection, or lead migration occurred. He continued to report more than 90% pain relief at his two- and five-month follow-up visits, with marked improvement in daily function and decreased opioid requirements, reducing hydrocodone-acetaminophen use from one-half tablet twice daily to one-half tablet daily as needed, with some days requiring no opioid medication.

At his most recent visit, eight months following lead placement and six months after lead removal, he continued to experience durable benefit, reporting a VAS score of 4/10 and a Global Pain Scale score of 59.5.

## Discussion

In 1965, Melzack and Wall published the “gate control” theory of pain. They proposed that there is a gating mechanism within the spinal cord where the relative activity of small nociceptive and large sensory neurons determines which signals reach higher centers. By increasing the firing of large sensory neurons, this would “close the gate” and block nociceptive signals from reaching the brain. In 1967, Wall and Sweet applied this theory experimentally in cats and tested it on their own infraorbital nerves, representing one of the earliest applications of PNS. In the same year, Shealy and Mortimer described their analgesic results with dorsal column SCS using a modified electrical stimulator initially designed to stimulate the carotid sinus [12].

Although the exact mechanism of action of PNS is unknown, evidence has shown that central and peripheral mechanisms contribute to the analgesic effect. It has been theorized that stimulation of non-nociceptive, large-diameter, low-threshold Aβ fibers increases the activity of inhibitory dorsal horn interneurons involved in the processing and transmission of pain from the Aδ and C fibers. Inhibiting pain-signal transmission at the spinal cord level prevents propagation to the central nervous system (CNS). PNS has also been theorized to decrease central sensitization and hyperalgesia by reducing excessive peripheral nociceptive activity in the spinal cord. This results in inhibition of wide dynamic range (WDR) neurons in the dorsal horn and decreased Aβ fiber-induced activity in the medial lemniscal pathway in the brain [9,13].

Historically, PNS was adapted from SCS, as no devices were designed specifically for the periphery. When PNS was applied in those rare cases, SCS systems were used peripherally. Huntoon et al. described an ultrasound-guided percutaneous approach to PNS lead placement, negating the need for a surgical cutdown approach [12]. Eventually, specifically designed open-coil leads for PNS were developed with axial flexibility to enable tissue ingrowth within the coil and secure the leads. These advancements have led to increased use of temporary percutaneous PNS (up to 60 days) to treat a wide variety of pain conditions [13]. The United States Food and Drug Administration (FDA) has cleared PNS systems for up to 60 days of use. This ability to provide electrical stimulation therapy for extended periods may serve as definitive treatment and possibly decrease the need for permanently implanted systems [12,14]. Evidence supporting the use of PNS includes prospective clinical trials, including multiple randomized controlled trials showing improvement in pain and sustained relief following treatment completion and lead removal [9]. Real-world studies have shown similar results. Huntoon et al. published a retrospective analysis of 6,100 patients, reporting that 71% experienced at least 50% pain relief and/or improvement in quality of life at the end of the 60-day PNS treatment, with similar outcomes across nerve targets [14]. Pingree et al. also published real-world data from a cross-sectional follow-up survey of 252 patients, with 50% (125/252) reporting ≥50% reductions in pain and/or improvement in quality of life compared with baseline. Of those who were end-of-therapy responders, 61% (112/185) continued to report sustained improvement (at least 50% pain relief and/or improvement in quality of life) at the time of survey completion, which ranged from three to 30 months from the start of treatment [15].

Despite the limited evidence base for neuropathic pain of the trunk, which includes TAP PNS [9], our findings suggest it may be a safe and effective option for the management of chronic refractory abdominal pain. By modulating the signals of the anterior rami of the T6-L1 spinal nerves, which provide sensation to the entire anterolateral abdominal wall, we report improved pain control and functional improvement along with reductions in daily MME. These cases support the feasibility of TAP PNS as a treatment option for chronic refractory abdominal pain. As a case report, there are multiple limitations that restrict the strength of our findings. The small sample size and retrospective nature limit the ability to draw causal inferences. Follow-up was limited, preventing assessment of long-term durability. This case report highlights the potential utility of TAP PNS and raises further questions. Larger prospective studies are needed to evaluate long-term efficacy, optimal patient selection, and optimal lead positioning.

## Conclusions

This case report of two patients demonstrates that targeting the segmental nerve branches within the TAP with PNS may offer an effective, minimally invasive treatment for chronic abdominal pain of varied etiologies when failure of multiple prior medications and interventions occurs. One patient had bilateral ilioinguinal neuralgia and another had post-herpetic neuropathic abdominal wall pain; both achieved substantial and sustained pain relief and functional improvement. These cases show that although different in underlying pathology, both responded to stimulation of the anterior rami within the TAP. In the one case where the patient was prescribed oral opioids, they were able to reduce opioid requirements following TAP PNS. These outcomes underscore the feasibility and therapeutic potential of TAP PNS across different abdominal pain syndromes and are consistent with case reports in the literature showing reduced pain with placement of PNS leads within the TAP. Further research is warranted to define optimal patient selection, lead placement, and long-term durability, but these findings support ongoing evaluation of TAP PNS as a valuable addition to the spectrum of peripheral neuromodulation therapies.
